# Novel regulatory roles of omega-3 fatty acids in metabolic pathways: a proteomics approach

**DOI:** 10.1186/1743-7075-11-6

**Published:** 2014-01-17

**Authors:** Abeer A Ahmed, Kayode A Balogun, Natalia V Bykova, Sukhinder K Cheema

**Affiliations:** 1 Department of Biochemistry, Memorial University of Newfoundland, St. John’s, NL, A1B 3X9, Canada; 2 Department of Biology, Memorial University of Newfoundland, St. John’s, NL, Canada; 3 Current address: Agriculture and AgriFood Canada, Winnipeg, MB, Canada

## Abstract

**Background:**

Omega-3 polyunsaturated fatty acids (*n*-3 PUFA) have been shown to alleviate the symptoms of metabolic disorders, such as heart disease, diabetes, obesity and insulin resistance. Several putative mechanisms by which *n*-3 PUFA elicit beneficial health effects have been proposed; however, there is still a shortage of knowledge on the proteins and pathways that are regulated by *n*-3 PUFA.

**Methods:**

Using two dimensional polyacrylamide gel electrophoresis (2D-PAGE) and liquid chromatography-tandem mass spectrometry (LC-MS/MS) analysis, we investigated the effects of diets high or low in *n*-3 PUFA on hepatic proteomic profile of C57BL/6 mice.

**Results:**

The findings show for the first time that high dietary *n*-3 PUFA reduced the expression of regucalcin, adenosine kinase and aldehyde dehydrogenase. On the other hand, diets high in *n*-3 PUFA increased the expression of apolipoprotein A-I, S-adenosylmethionine synthase, fructose-1, 6-bisphosphatase, ketohexokinase, malate dehydrogenase, GTP-specific succinyl CoA synthase, ornithine aminotransferase and protein disulfide isomerase-A3.

**Conclusions:**

Our findings revealed for the first time that *n*-3 PUFA causes alterations in several novel functional proteins involved in regulating lipid, carbohydrate, one-carbon, citric acid cycle and protein metabolism, suggesting integrated regulation of metabolic pathways. These novel proteins are potential targets to develop therapeutic strategies against metabolic disorders.

## Background

Nutrients have the ability to interact and modulate molecular mechanisms responsible for an organism’s physiological functions [1]. Over the past decades, the importance of nutrients, especially omega (*n*)-3 polyunsaturated fatty acids (PUFA), in the aetiology of chronic diseases has gained a lot of attention [2-4]. *N*-3 PUFA have been shown to be beneficial in a variety of disease conditions such as, atherosclerosis [5], obesity [6], and obesity induced insulin resistance in liver [7,8]. The cardio-protective effects of *n*-3 PUFA are the most characterized; *n*-3 PUFA improve cardiovascular health mainly by reducing plasma triglyceride (TG) levels [9], resolving the symptoms of arrhythmia [10] and inflammation [11]. The *n*-3 PUFA cardio-protective effect depends mainly on its dosage [12-14]. The American Heart Association has recommended 1,000 mg/d for treatment of existing cardiovascular diseases (CVDs) [15]. Studies have also shown that *n*-3 PUFA stimulate muscle glycogen synthesis [16] and modulate antioxidant enzyme activity, such as superoxide dismutase and catalase in livers from diabetic rats fed a high fat diet [17]. Conversely, diets high in *n*-6 PUFA are considered to increase the risk of inflammatory [18] and oxidative stress pathways [19], which are confounding factors of most metabolic diseases. Several mechanisms have been proposed by which *n*-3 and *n*-6 PUFA exert their biological actions [20,21], however, the information available is scanty, inconclusive, and does not explicitly define the proteins and pathways involved in the actions of PUFA.

*N*-3 and *n*-6 PUFA form bioactive mediators which act on different receptors and proteins in the body [22]. Proteins are important mediators of biological activities in all living cellular units, and the proteome is comprised of all expressed proteins encoded by the genome of a cellular system [23]. The analysis of nutrient mediated changes in the proteomic profile can lead to identification of novel proteins involved in the regulation of metabolic pathways [24]. In the current study, we used 2D-PAGE coupled with LC-MS/MS to investigate the effect of *n*-3 PUFA on the regulation of hepatic proteomic profile of C57BL/6 mice. Our findings show for the first time that dietary *n*-3 PUFA reduced the expression of regucalcin, a key molecule involved in metabolic disorders including diabetes and lipid metabolism. We also report for the first time that *n*-3 PUFA regulate several proteins involved in the regulation of lipid metabolism, one-carbon metabolism, carbohydrate, citric acid cycle, and protein synthesis.

## Methods

### Diet and animals

A base semi-synthetic diet designed specifically to permit the control of fat level at 20% w/w was obtained in powdered form with the fat source omitted (MP Biomedicals, OH, USA). Menhaden oil (Sigma-Aldrich, MO, USA); lard, safflower oil and extra-virgin olive oil were used to prepare two different oil mixtures containing 10% (high *n*-3) and 2% (low *n*-3) *n*-3 PUFA of the total fat and has been previously published [25]. The amount of saturated fatty acids (SFA), monounsaturated fatty acids (MUFA) and total PUFA was kept constant. The two experimental diets differed only in the *n*-3 PUFA amount; the high *n*-3 PUFA diet contains 10% *n*-3 PUFA while the low *n*-3 PUFA diet contains 2% *n*-3 PUFA of the total dietary fat. Gas–liquid chromatography (GLC) was used to determine the fatty acid composition of the diet, which has been previously published [25]. Seven weeks old C57BL/6 mice were purchased from Charles River Laboratories (MA, USA). Animals were housed in a temperature-controlled animal facility with a 12 h light–dark cycle. The mice were maintained for one week acclimatization period on a regular chow diet (Prolab RMH 3000) purchased from PMI nutrition (MO, USA). Female mice were placed on one of the two experimental diets (low or high *n*-3 PUFA diets) for 2 weeks prior to mating, and they continued on designated diets through gestation, lactation, and until weaning. At weaning, male and female offspring were continued on their mothers’ diets for four months. Animals were provided with water and fresh diet ad libitum, every other day. Body weights were recorded once a week, and diet intake was recorded every other day. No significant differences were observed in both body weight and diet intake (Data not shown). At the end of the experimental period, animals (n = 4) were fasted overnight and blood was collected by cardiac puncture in tubes containing EDTA (4.5 mM, pH 7.4) to separate plasma. Livers were immediately snap frozen in liquid nitrogen and stored at −80°C until further use. Institutional Animal Care Committee at Memorial University approved all experiments in compliance with the guidelines of the Canadian Council for Animal Care.

### Lipids and glucose analysis

Hepatic lipids were extracted according to our previously published method [26], and analyzed for TG concentrations using a TG assay kit (# 236–60, Sekisui Diagnostics, P.E.I Inc., Canada). Plasma samples were used for determination of non-esterified fatty acids (NEFA) using a kit (# 993–35191, Wako Chemicals Inc., USA). Fasting blood glucose concentrations were measured at the time of sacrifice using a commercially available glucometer (Lifescan Inc. CA, USA) after snipping the tail.

### Ornithine aminotransferase (OAT) activity analysis

Hepatic OAT activity was assayed according to the method of Herzfeld and Knox [27] and protein concentration of liver extract was determined using the Biuret method [28]. The OAT enzyme activity was standardized for linearity with time, protein concentration, and was expressed as μmol/min/g liver.

### Proteomic profiling

Liver samples (n = 4 per treatment group) (30 mg each) were homogenized in 40 mM Tris to profile the hydrophilic proteome soluble in aqueous buffer. Separation of the supernatant was performed after centrifugation (3000 rpm for 3 minutes at 4°C). Protein concentration was assayed by the method of Bradford [29]. An aliquot of 800 μg total protein was added to 10 ml pre-chilled 85% acetone/0.07% dithiothreitol (DTT) at −20°C for a final concentration of 80% acetone; washing and centrifugation steps were repeated six times to remove all the water soluble contaminants, and the pure protein pellet was dried under nitrogen gas. Washing with several acetone precipitation rounds improves the quality of Isoelectric focusing (IEF) significantly [30]. The purified protein sample was suspended directly into rehydration buffer (7.0 M urea, 2.0 M thiourea, 4% CHAPS, 30 mM DTT, 1% Bio-Rad 3–10 ampholyte, bromophenol blue) to a final volume of 500 μL per sample, and centrifuged at 100,000 × g for 30 min prior to overnight loading via rehydration of immobilized pH gradient (IPG) strips. IEF was conducted with the Ettan IPGphor II system and Manifold tray (GE Healthcare, Piscataway, NJ, USA) using 24 cm ReadyStrip IPG (Bio-Rad, Mississauga, Ontario Cat. No.163-2042). The strips were focused for a total of 100 kVh prior to running the second dimension gels. The IPG strips were incubated for 10 min in equilibrium buffer (1.5 M Tris–HCl, pH 8.8, sodium dodecyl sulfate (SDS), urea, glycerol, bromophenol blue) with 1% DTT followed by 10 min in the same buffer containing 2.5% iodoacetamide. The second-dimension Tris-glycine SDS-PAGE was carried out using 20 cm gradient 10–20% acrylamide gels as described elsewhere using an Ettan DALT six (GE Healthcare) apparatus [31]. The total protein content was detected by staining overnight with 0.15% w/v Coomassie blue R250; destained for 1 h with 25% v/v ethanol, 7% v/v acetic acid.

### Protein quantification and identification using progenesis gel imaging

The images of the total protein pattern of 2D-gels were captured by video imaging using the ImageScanner III (LabScan 6.0 software, GE Healthcare, Life Sciences, Sweden). We then used a specialized software, Progenesis Samespots, version 3.1 (Non-linear Dynamics, Newcastle upon Tyne, UK) to align and quantify the protein spots from captured 2D-gel images. The use of identical spot boundaries across all gels, background subtraction, and normalization to total staining intensity in each gel ensured comparable data between all gels with the Progenesis Samespots software. Each spot was manually evaluated using a 3D image display to exclude artifacts (spiky or irregularly shaped spots, split spots etc.). For the identified real spots, image realigning, noise filtering, and spots segmentation were carried out using default setting [32]. Automatic analysis was performed on all the aligned images using the analysis wizard. The aligned images were grouped into high and low *n*-3 PUFA group, and the statistically ranked list of spots was evaluated.

### In-gel digestion and peptide extraction

Excised protein spots in 1.5 siliconised polypropylene vials were washed 3 times with 100 mM ammonium bicarbonate/acetonitrile (NH_4_HCO_3_/ACN) and proteins were reduced with 10 mM DTT in 100 mM NH_4_HCO_3_ for 30 min at 56°C [33]. After cooling to room temperature, excess DTT solution was removed, and samples were alkylated with 55 mM iodoacetamide in 100 mM NH_4_HCO_3_ for 30 min at room temperature in the dark. Excess iodoacetamide solution was removed, and gel pieces were washed again with 100 mM NH_4_HCO_3_/ACN, 1:1 v/v. The samples were dehydrated for 20 min with ACN, and subsequently rehydrated with 12.5 ng/μl sequencing grade modified Trypsin buffer (100 mM NH_4_HCO_3_, 10% ACN, 2.5 mM CaCl_2_) for 30–40 min on ice [34]. Samples were incubated at 37°C overnight. After cooling to room temperature, 50 μl of 5% formic acid (FA) was added, samples were vortexed and centrifuged at 6000 rpm for 2 min, and the clear supernatant was collected into fresh siliconised tubes. The extraction process was repeated using 1% FA, 5% ACN; 1% FA, 60% ACN; 1% FA and 99% ACN. The combined extracts were dried under vacuum.

### Mass spectrometric analysis of peptide extracts and database searching

For peptide mapping and fragmentation analysis with matrix assisted laser desorption/ionization time-of-flight mass spectrometry (MALDI TOF MS), each concentrated peptide extract was purified with a μC18-Ziptip (Millipore, Billerica, MA) pipette tip. The bound peptides in the ZipTip were eluted using the elution solution (50% ACN in 5% FA, 2,5-Dihydroxybenzoic acid (DHB 10 mg/67 μL). For sample spotting, we used the dried droplet method [35]. 4 μL sample was deposited on the target into two spots with 2 μL each. Calibration was done first externally to obtain 10–20 ppm accuracy with known peptide standards. Internal calibration was performed with known matrix cluster signals and tryptic autolysis peptide signals to achieve a mass accuracy of less than 100 ppm. Single MS with m/z range 600–3000, and MS/MS analysis was conducted using the QSTAR XL hybrid quadrupole/time of flight mass spectrometer equipped with an o-MALDI ion source (Applied Biosystems, Foster City, CA) located at Memorial University Genomics and Proteomics (GaP) facility. The instrument exhibits a mass resolving power of 10 000 full-width half-maximum, and accuracy within a few millidaltons in the TOF spectra in both MS and MS/MS modes. A peak list was created with the Applied Biosystems Data Explorer from the unsmoothed raw data spectrum after de-isotoping. Monoisotopic m/z values from known matrix-cluster peaks and signals from trypsin autolysis peptides or known peptides derived from keratins [36] were manually removed from this list before it was copied to the query section of the MASCOT Peptide Mass Fingerprint online entry form at http://www.matrixscience.com[37]. The following settings were applied for the online database search: Database = NCBInr 20111022 (15670863 sequences; 5387755057 residues); Taxonomy = Musculus; Enzyme = Trypsin (with a maximum of 2 missed cleavages); Fixed modifications = Carbamidomethyl (C); Variable modifications = Acetyl (Protein N-term) and Oxidation (M); Peptide tolerance = ±100 ppm; Mass values = MH + (monoisotopic). Only probability scores, p < 0.05, were considered.

### Protein identification using liquid chromatography-tandem mass spectrometry

Separation of peptide mixture was conducted using a DIONEX UltiMate 3000 Nano LC System (Germering, Germany). Protein digest (250 fmol) was loaded onto a C18 pre-column (LC Packing, Sunnyvale, CA) for desalting and concentrating. Peptides were then eluted from the pre-column and separated on a nano-flow analytical C18 column (PepMap 75 μm i. d., LC Packing, Sunnyvale, CA) at 180 nl/min using an ACN gradient. The mobile phase consisted of (A) 0.1% FA/0.01% trifluoroacetic acid (TFA)/2% ACN and (B) 0.08% FA/0.008% TFA/98% ACN. A gradient of 0% B for 10 min, 0-60% B for 55 min, 60-90% for 3 min, 90% B for 5 min was applied. An Applied Biosystems QSTAR XL (Applied Biosystems/MDS Sciex, Foster City, USA) hybrid quadrupole TOF-MS/MS system equipped with a nanoelectrospray source (Protana XYZ manipulator) located at Memorial University Genomics and Proteomics (GaP) facility was used for LC-MS/MS peptide sequence analysis. The nanoelectrospray was generated from a PicoTip needle (10 μm i.d., New Objectives, Wobum, USA) at a voltage of 2400 V. Information-dependent acquisition in positive ion mode was employed with one MS m/z range of 400–1500 and MS/MS of three most abundant ions with charge state 2 to 4 in each cycle, 60 s dynamic exclusion with a mass tolerance of 100.000 ppm, using Analyst® QS 1.1 software (Applied Biosystems/MDS Sciex, Foster City, USA). The fragment intensity multiplier was set to 20 and maximum accumulation time was 3 s. Spectra acquired were submitted for peak list generation by Mascot Distiller integrated into Analyst® QS 1.1 software (Applied Biosystems/MDS Sciex, Foster City, USA). The peptide tandem mass spectra were searched against the NCBI non-redundant protein database 20120722 (19256848 sequences; 6606790375 residues) using MASCOT search engine with a precursor mass tolerance of 0.2 Da and a fragment ion mass tolerance of 0.2 Da. Peptides were considered identified if the Mascot score was over the 95% confidence limit based on the ‘identity’ score of each peptide. Mass lists in the form of Mascot Generic Files were used as input for Mascot MS/MS ion searches of the NCBInr database using the matrix science web server (http://www.matrixscience.com) [37]. The following settings were applied for the online database search: Database = NCBInr; Taxonomy = Musculus; Enzyme = Trypsin (with a maximum of 2 missed cleavages); Fixed modifications = Carbamidomethyl (C); Variable modifications = Acetyl (Protein N-term) and Oxidation (M); Peptide tolerance = ±100 ppm; Mass values = MH + (monoisotopic). Only probability scores, p < 0.05, were considered.

### Statistical analysis

The results for biochemical parameters were analysed using unpaired t-test (GraphPad software Inc., CA). Results were expressed as mean ± standard deviation (SD). Differences were considered to be statistically significant if the associated p value was < 0.05.

## Results and discussion

### Proteomic profile

**Figure 1:**
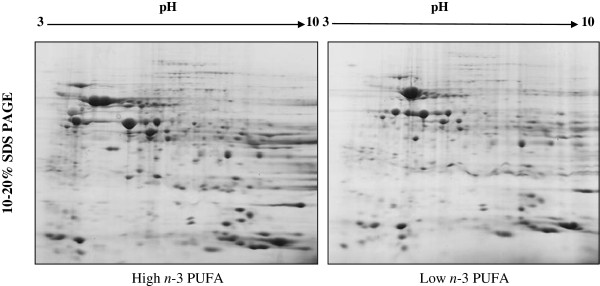
**2D**-**PAGE image of whole hepatic proteome of mice fed high or low *n*-3 polyunsaturated fatty acid enriched diets.** The images represent scanned Coommasie stained gels of female C57BL/6 mice. Proteins were separated in the first dimension using immobilized pH 3–10 strips and then separated by mass using 10 to 20% polyacrylamide gradient gels. Over 275 spots were resolved and detected using Progenesis Samespots, version 3.1.

The separation of proteins by 2D-PAGE resolved approximately 275 proteins that could be detected and quantified by automated matching using the specialized software Progenesis Samespots, version 3.1 (Non-linear Dynamics, Newcastle upon Tyne, UK) (Figure 1). A comparison of female mice fed with high and low *n*-3 PUFA diets revealed 59 spots that were different, with a fold difference in the range of 2–9 folds. Differentially expressed proteins in females fed high or low *n*-3 PUFA were grouped according to their metabolic functions in the following pathways: lipid metabolism, one-carbon metabolism, carbohydrate, citric acid cycle and protein synthesis.

### *N*-3 PUFA alters regucalcin and ApoA-I expression to regulate lipid metabolism

**Figure 2:**
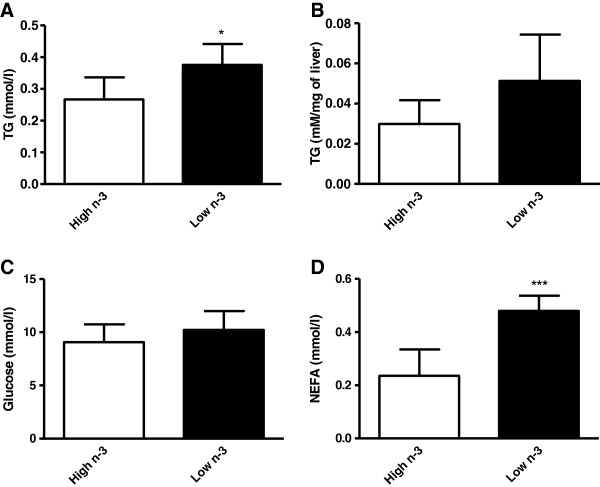
**Biochemical parameters of female mice fed high or low *n*-3 polyunsaturated fatty acid enriched diets.** Concentrations of plasma triacylglycerol (TG) **(A)**, hepatic TG **(B)**, plasma glucose **(C)**, and plasma non-esterified fatty acids (NEFA) **(D)** were measured as described in the methods section. Values are expressed as means ± SD (n = 8), and P-value was calculated using unpaired t-test. *P < 0.05, ***P < 0.0001.

Mice fed a high *n*-3 PUFA diet showed a lower expression of regucalcin compared to mice fed a low *n*-3 PUFA diet (Table 1). Regucalcin is mainly expressed in rodent liver, and higher expression of regucalcin is linked to metabolic disorders including diabetes and lipid metabolism [38,39]. Regucalcin has been found to play a multifunctional role in different tissues [40], and is primarily involved in the maintenance of intracellular Ca^2+^ homeostasis [39]. Regulation of calcium homeostasis is important in many metabolic pathways including glucose metabolism and diabetes [41,42]. There are evidences linking higher expression of regucalcin to adipogenesis in adipocytes [43], and also alterations in lipid and glucose metabolism in vivo [44], which are predisposing factors to obesity and diabetes. Overexpression of regucalcin was also found to enhance glucose utilization and lipid production in the cloned rat hepatoma H4-II-E cells in vitro [44,45]. Concomitant with a decrease in regucalcin on a high *n*-3 PUFA diet was a significant decrease in plasma TG concentrations as compared to mice fed a low *n*-3 PUFA diet (Figure 2A). Although feeding a high *n*-3 PUFA diet had no significant effect on hepatic TG concentration, there was a trend towards a decrease compared to mice fed a low *n*-3 PUFA diet (Figure 2B). These findings suggest that the regulation of lipid metabolism by *n*-3 PUFA is likely mediated via regucalcin, which is a novel finding. We also observed a higher expression of Apolipoprotein A-I (ApoA-I) in mice fed the high *n*-3 PUFA diet. ApoA-I is the major protein component of HDL-c that mediates reverse cholesterol transport from extra-hepatic tissues to the liver for excretion [46]. It has been reported that *n*-3 PUFA supplementation alters lipoprotein containing proteome and suggests that this alteration will improve the functionality of HDL particle [47]. Increasing ApoA-I levels is an attractive strategy for the prevention of CVDs [3], thus a diet high in *n*-3 PUFA is likely inducing cardioprotective effects via increasing ApoA-I levels.

**Table 1:** **Hepatic proteins identified via peptide mapping and MS**/**MS fragmentation in C57BL**/**6 mice fed a diet high or low in *n***-**3 polyunsaturated fatty acids**

**Protein**	**GI**	**Mass**	**Score**	**Matches**	**Sequences**	**Metabolic pathway**
**Fructose-1,6-biphosphatase**	51036635	40040	463	6 (6)	6 (6)	**Gluconeogenesis**
**Ketohexokinase**	31982229	33290	348	5 (4)	5 (4)	
**Apolipoprotein A-I**	109571	30358	295	7 (2)	7 (2)	**Lipid transport and metabolism**
**Cytosolic malate dehydrogenase**	387129	36625	238	4 (4)	4 (4)	**Citric acid cycle**
**GTP-specific succinyl COA synthase beta subunit**	3766203	44115	393	7 (5)	7 (5)	**Citric acid cycle**
**6-phosphogluconolactonase**	13384778	27465	501	10 (6)	10 (6)	**Pentose phosphate pathway**
**Regucalcin**	6677739	33899	90	2 (1)	2 (1)	**Regulatory protein**

GI=GenBank sequence identification number.

### *N*-3 PUFA regulate proteins involved in one-carbon metabolism

Mice fed a high *n*-3 PUFA diet showed a higher expression of S-adenosylmethionine synthase (SAM synthase) (Table 2) compared to mice fed a low *n*-3 PUFA diet. SAM synthase [48] catalyzes the synthesis of SAM from methionine and is responsible for regeneration of methionine from homocysteine (Hcy) [49]. Elevated plasma total Hcy levels are a fairly well established risk factor for CVD [50]. A high-fat diet was found to increase plasma Hcy levels in C57BL/6 mice [51], whereas *n*-3 PUFA was negatively associated with plasma Hcy [52]. A meta-analysis study also showed that the high consumption of *n*-3 PUFA decreases plasma Hcy [53]. Our study revealed that increased SAM synthase in mice fed high *n*-3 PUFA diet could be responsible for lowering Hcy levels.

In addition to increasing the expression of SAM synthase, a diet high in *n*-3 PUFA caused a decrease in adenosine kinase (ADK) protein expression compared to mice fed a low *n*-3 PUFA diet. ADK plays an important role in regulating the intracellular as well as extracellular concentrations of adenosine by catalyzing the phosphorylation of adenosine to AMP through the use of ATP [54]. ADK is highly expressed in the liver [55], and has been shown to play an important role in methyltransferase reactions to control SAM and Hcy levels [56]. A recent study has reported that inhibition of ADK promotes rodent and porcine islet beta-cell replication, and the authors have proposed ADK inhibition as a strategy for the treatment of diabetes [57]. Thus inhibition of ADK by *n*-3 PUFA could be a potential dietary based therapeutic strategy under diabetic conditions. We did not find a significant effect of the diets on plasma glucose levels (Figure 2C), however the levels of NEFA were significantly lower in animals fed a high *n*-3 PUFA diet (Figure 2D), supporting insulin sensitizing effects of *n*-3 PUFA.

### Effect of *n*-3 PUFA on the regulation of carbohydrate metabolism

*N*-3 PUFA have also been shown to increase insulin sensitivity [58] via regulating proteins involved in carbohydrate metabolism [59]. We observed a higher expression of hepatic fructose-1, 6-bisphosphatase (FBPase) and ketohexokinase (KHK) in mice fed a high *n*-3 PUFA diet (Table 1). FBPase converts fructose-1, 6-bisphophate to fructose-6-phosphate and is considered a key regulatory enzyme in gluconeogenesis [60]. A diet rich in *n*-3 PUFA was previously shown to increase FBPase expression in liver of 3 day old rat pups [61]. Fructose-6-phosphate is metabolised to glucose-6-phosphate thereby acting as a substrate for pentose phosphate pathway (PPP) [62]. We observed that mice fed a high *n*-3 PUFA diet had higher expression of 6-phosphogluconolactonase (6PGL) compared to mice fed the low *n*-3 PUFA diet (Table 1). 6PGL catalyzes the second step in the PPP [63], which is a major source of NADPH and pentose sugars necessary for oxidative stress defence and nucleotide synthesis [64]. There are two distinct phases in this pathway; the first is the oxidative phase, which generates NADPH, and the second phase is the non-oxidative synthesis of 5-carbon sugar [65]. NADPH that results from the PPP is mainly used in reductive biosynthesis reactions within cells through increasing glutathione reductase, which is necessary to regenerate glutathione [64]. Both epidemiological and animal studies have also shown that high fructose predisposes to type-II diabetes and metabolic syndrome [66-68]. We found that mice fed a high *n*-3 PUFA diet had higher expression of KHK, which initiates the pathway to metabolize dietary fructose [69]. *N*-3 PUFA has previously been reported to have beneficial effects under fructose induced diabetic conditions [70]. This is the first report that provides evidence for *n*-3 PUFA mediated increase in fructose metabolism, likely due to an increase in the protein expression of KHK.

### Effect of *n*-3 PUFA on proteins involved in citric acid cycle

**Figure 3:**
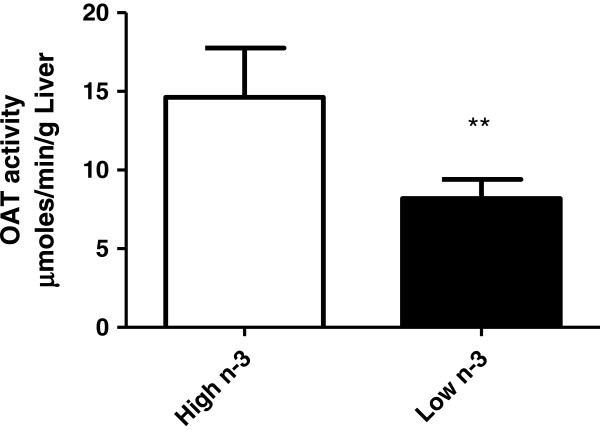
**The effect of high and low *n*-3 polyunsaturated fatty acid enriched diets on hepatic ornithine aminotransferase (OAT) enzyme activity.** The OAT enzyme activity was measured according to details given under the methods section. The enzyme activity was expressed as μmol/min/g liver; values are mean ± SD (n = 4 per treatment).

Mice fed a high *n*-3 PUFA diet showed higher protein expression of cytosolic malate dehydrogenase (MDH) and GTP specific succinyl CoA synthase (SCS) beta subunit compared to mice fed a low *n*-3 PUFA diet (Table 1). MDH is an enzyme that reversibly catalyzes the oxidation of malate to oxaloacetate using the reduction of NAD + to NADH [71], and is also involved in gluconeogenesis. *N*-3 PUFA was previously found to regulate the metabolic function of liver effectively by increasing MDH enzyme activity of rat liver [59,72]. SCS catalyzes the reversible reaction of succinyl-CoA to succinate, and is the only mitochondrial enzyme capable of ATP production via substrate level phosphorylation in the absence of oxygen [73]. SCS plays a key role in the citric acid cycle, ketone metabolism and heme synthesis [74]. Upregulation of MDH and SCS in the high *n*-3 PUFA diet group would result in an increase in oxaloacetate levels which has several possible fates: 1) transamination to aspartate, 2) conversion into glucose by the gluconeogenic pathway, 3) condensation with acetyl CoA to form citrate, or 4) conversion into pyruvate. Moreover, the animals fed a high *n*-3 PUFA diet revealed a dramatic increase in the expression of OAT compared to mice fed a low *n*-3 PUFA diet. We measured OAT enzyme activity to confirm the effect of dietary *n*-3 PUFA and observed a significant increase (p = 0.0086) in OAT enzyme activity in animals fed a high *n*-3 PUFA diet compared to animals fed the low *n*-3 PUFA diet (Figure 3). This is the first study to report that *n*-3 PUFA enriched diets significantly increased OAT protein expression and enzyme activity. OAT is a pyridoxal-50-phosphate-dependent mitochondrial matrix aminotransferase that catalyses the inter-conversion of ornithine into glutamate semi-aldehyde [75]. OAT is located at a crossing between two important metabolisms: arginine and polyamine metabolism on one side and glutamate and proline metabolism on the other side [76]. OAT is mainly found in the liver where its response to hormones and variations in dietary protein intake is subject to complex regulatory mechanisms [27,77,78]. OAT plays a role in the adaptation to the level of protein supply, and also to the regulation of the availability of arginine and glutamine. Glutamine has a number of important regulatory roles in increasing protein synthesis, decreasing protein degradation, and also regulating arginine functions (i.e. stimulating the release of growth hormones, insulin-like growth factor 1, insulin and prolactin) [79]. Thus, an overexpression of OAT could lead to changes in protein metabolism, thereby decreasing insulin resistance in type II diabetes. To the best of our knowledge, this is the first study that establishes an association between *n*-3 PUFA and hepatic OAT expression. Taken together, high *n*-3 PUFA diet appears to regulate carbohydrate metabolism through alteration of many functional proteins such as FBPase, KHK, MDH, SCS and OAT.

### Effect of *n*-3 PUFA on the regulation of protein synthesis

Animals fed a diet high in *n*-3 PUFA revealed an increase in protein disulfide isomerase-A3 (PDI-A3) expression compared to mice fed a low *n*-3 PUFA diet. PDI-A3 constitutes a family of structurally related enzymes that catalyze disulfide bond formation, reduction, or isomerization of newly synthesized proteins in the lumen of the endoplasmic reticulum [80]. It has both a disulfide isomerase activity that helps the correct formation of disulfide bridges between cysteine residues and a chaperone activity preventing proteins from misfolding in the endoplasmic reticulum [81]. A reduction in PDI-A3 activity is associated with impaired liver function [82]. Previously, it has been reported that diets enriched in menhaden oil increased activation (phosphorylation) of anabolic signaling proteins in muscle during administration of insulin and amino acids [83], and also increased the non-oxidative whole-body disposal of amino acids [83,84]. *N*-3 PUFA derived resolvins and protectins also prevent liver DNA damage and oxidative stress [85]; thus, significantly decreasing inflammatory liver injury and hepatic steatosis [86,87]. Our findings suggest that diets enriched in *n*-3 PUFA increase protein synthesis through inducing the expression of disulfide isomerase-A3.

We also observed a lower expression of aldehyde dehydrogenase (ALDH) in mice fed the high *n*-3 PUFA diet compared to mice fed the low *n*-3 PUFA diet (Table 2). ALDHs are the products of a large gene family and catalyze irreversible oxidation of a variety of biological aldehydes including products of lipid peroxidation [88]. Increased levels of ALDH are indicative of oxidative stress [89]. The *n*-3 PUFAs were shown to decrease oxidative stress [90,91], and also to reduce the vascular-derived oxidative stress associated with diabetes [92], which are likely mediated via inhibition of ALDHs. These findings were further supported by increased expression of lactoylglutathione lyase (glyoxalase 1) in mice fed a high *n*-3 PUFA diet (Table 2). Glyoxalase 1 is critical for the detoxification of reactive dicarbonyls, such as methylglyoxal [93]. These reactive dicarbonyls are potent precursors of advanced glycation end products (AGEs), well known to be increased under diabetic conditions [94,95]. An increased expression of glyoxalase 1 in high *n*-3 PUFA diet would therefore cause a reduction in AGE production, thereby eliciting beneficial health effects under diabetic conditions.

**Table 2:** **Fold differences in abundance of hepatic proteins identified by LC**-**MS**/**MS in C57BL**/**6 mice fed a diet high or low in *n***-**3 polyunsaturated fatty acids**

**#Protein**	**Function**	**High *n***-**3 PUFA**	**Low *n***-**3 PUFA**	**GI**	**Mass**	**Score**	**Matches**
**Ornithine aminotransferase**	Nitrogen homeostasis	28.25↑		8393866	48723	60	2 (1)
**S**-**adenosylmethionine synthase**	One-carbon metabolism	1.88↑		19526790	44051	503	11 (8)
**Disulfide isomerase**-**A3**	Protein folding	9.18↑		351707448	57366	81	2 (1)
**Aldehyde dehydrogenase**	Energy production		1.43↑	560645	56686	55	1 (1)
**Adenosine kinase**	Phosphorylation		1.66↑	19527306	40466	379	12 (2)
**Lactoylglutathione lyase**	Detoxification	1.69↑		165932331	20967	278	7 (6)

The images of the total protein pattern of 2D-gels were captured by video imaging using the ImageScanner III (LabScan 6.0 software, GE Healthcare, Life Sciences, Sweden). Progenesis Samespots, version 3.1, a specialized software (Non-linear Dynamics, Newcastle upon Tyne, UK) was then used to align and quantify the protein spots from the captured 2D-gel images. PUFA, polyunsaturated fatty acids; GI, GenBank sequence identification number; ↑= upregulation.

## Conclusions

**Figure 4:**
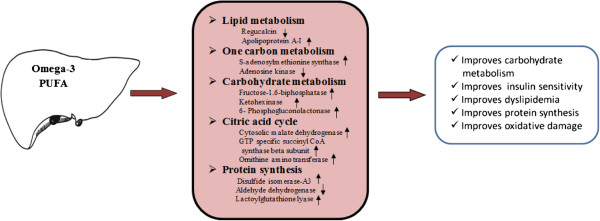
**Schematic representation of the effects of *n*-3 polyunsaturated fatty acids (PUFA) on the regulation of metabolic pathways.** High *n*-3 PUFA feeding caused an alteration in metabolic pathways; lipid metabolism, one-carbon metabolism, carbohydrate, citric acid cycle and protein synthesis.

In conclusion, our data suggests an important functional role of dietary *n*-3 PUFA in regulating proteins involved in lipids, glucose metabolism and protein synthesis as illustrated in Figure 4. We are reporting for the first time that *n*-3 PUFA down-regulates the expression of regucalcin, a potent player in lipid metabolism disorders. Furthermore, we have been able to demonstrate a novel involvement of n-3 PUFA in the regulation of proteins involved in one-carbon metabolism (SAM synthase and ADK), carbohydrate metabolism (FBPase, KHK and 6PGL), citric acid cycle (MDH, SCS and OAT), and protein synthesis (PDI-A3, ALDH and glyoxalase-1). Our findings have laid the foundation to undertake further studies to elucidate the potential health benefits of regulating the identified proteins and their pathways as a therapeutic strategy.

## Abbreviations

ADK: Adenosine kinase; AGEs: Advanced glycation end products; ALDH: Aldehyde dehydrogenase; CVDs: Cardiovascular diseases; DHB: 2,5-Dihydroxybenzoic acid; DTT: Dithiothreitol; FA: Formic acid; FBPase: Fructose-1, 6-bisphosphatase; GaP: Memorial University Genomics and Proteomics; GLC: Gas–liquid chromatography; Hcy: Homocysteine; IEF: Isoelectric focusing; IPG: Immobilized pH gradient; LC-MS/MS: Liquid chromatography-tandem mass spectrometry; MALDI TOF MS: Matrix assisted laser desorption/ionization time-of-flight mass spectrometry; MDH: Malate dehydrogenase; MUFA: Monounsaturated fatty acids; N-3 PUFA: Omega-3 polyunsaturated fatty acids; NEFA: Non-esterified fatty acids; NH4HCO3/CAN: Ammonium bicarbonate/acetonitrile; OAT: Ornithine Aminotransferase; PDI-A3: Protein disulfide isomerase-A3; PPP: Pentose phosphate pathway; 6PGL: 6-Phosphogluconolactonase; SAM: S-adenosylmethionine synthase; SCS: Succinyl CoA synthase; SD: Standard deviation; SDS: Sodium dodecyl sulfate; SFA: Saturated fatty acids; TFA: Trifluoroacetic acid; TG: Triglyceride; 2D-PAGE: Two dimensional polyacrylamide gel electrophoresis.

## Competing interests

The authors declare that they have no competing financial, professional or personal interests that might have influenced the performance or presentation of the work described in this manuscript.

## Authors’ contributions

AA conducted the 2D-PAGE experiments, in gel digestion of gel spots, analyzed and interpreted data, and drafted the manuscript; KB undertook the animal study, carried out the biochemical parameters, interpreted data and participated in manuscript preparation; NB provided open access to instrumentation needed for proteomics experiment and participated in final revisions, SKC conceived the study, initiated the experimental design, coordinated the study, interpreted data, and drafted the manuscript. All authors read and approved the final manuscript.
